# Effects of different levels of dried onion powder on nutrient digestibility, biochemical parameters, and nitrogen balance in Wistar albino rats with induced hyperuricemia

**DOI:** 10.3389/fphys.2023.1273286

**Published:** 2023-12-04

**Authors:** Muhammad Umer, Mahr Un Nisa, Nazir Ahmad, Muhammad Abdul Rahim, Fahad Al-Asmari

**Affiliations:** ^1^ Department of Nutritional Sciences, Faculty of Medical Sciences, Government College University, Faisalabad, Punjab, Pakistan; ^2^ Department of Food Science, Faculty of Life Sciences, Government College University, Faisalabad, Punjab, Pakistan; ^3^ Department of Food Science and Nutrition, Faculty of Medicine and Allied Health Sciences, Times Institute, Multan, Pakistan; ^4^ Department of Food and Nutrition Sciences, College of Agricultural and Food Sciences, King Faisal University, Al-Ahsa, Saudi Arabia

**Keywords:** onion, quercetin, hyperuricemia, growth performance, nitrogen balance, nutrient digestibility

## Abstract

**Introduction:** Onions (*Allium cepa* L.) are excellent sources of bioactive compounds and phytochemicals such as allicin, quercetin, fisetin, and other sulfurous compounds. Therefore, our study aimed to investigate the effects of dried onion powder on growth performance, nitrogen balance, and biochemical parameters in Wistar albino rats with induced hyperuricemia.

**Methods:** A total of 24 rats were randomly divided into four groups, with six in each group: HU (positive control) and HOT_1_, HOT_2_, and HOT_3_ groups, which received a diet containing onion powder at concentrations of 11.13, 14.84, and 18.61 g/100 g, respectively. Hyperuricemia was induced in rats by administering a new formulation intraperitoneally (250 mg/kg potassium oxonate) and orally (40 mg/kg potassium bromate) daily for 14 days. After confirmation of hyperuricemia induction, rats were fed with onion-treated diets with various concentrations of quercetin for 21 days.

**Results:** Significant decreases (*p* ≤ 0.05) in serum uric acid, alanine aminotransferase, aspartate aminotransferase, alkaline phosphatase, total bilirubin, total cholesterol, and low-density lipoprotein were observed. An increasing trend (*p* ≤ 0.05) in the levels of hemoglobin (Hb), white blood cell (WBC), red blood cell (RBC), and platelet count was observed. An improvement in the levels of serum high-density lipoprotein, triglycerides, blood urea nitrogen, serum creatinine, serum total protein and neutrophils, lymphocytes, and monocytes was observed. A positive progress (*p* ≤ 0.05) was observed in growth performance and nutrient digestibility.

**Conclusion:** In conclusion, a significantly lower uric acid level was observed in rats fed with HOT_2_ diet. Based on the ratio of the surface area (human/rat), the best recommended dose of onion for the incidence and prevention of hyperuricemia is 189.95 g, corresponding to the dose of 204 mg/day of quercetin in humans.

## 1 Introduction

Diet plays an important role in maintaining health by reducing the incidence of metabolic disorders (Jiang et al., 2021). Hyperuricemia is one of the metabolic disorders, which might occur due to the high level of purine and fructose and less water intake. Hyperuricemia is characterized by the elevated levels of serum uric acid, affecting 5%–30% of the population with increasing number of cases day by day (George and Minter, 2022). A recent study has shown an increase in the occurrence of hyperuricemia in Pakistan along with the burden of metabolic syndrome, weight gain, ischemic heart, and renal diseases (Qudwai and Jawaid, 2017). The most commonly considered method to control this disease is to inhibit the production of uric acid. Xanthine oxidase is a key enzyme found in the liver, and it is important for the catabolism of purines. It catalyzes the oxidation of hypoxanthine to xanthine and xanthine to uric acid. Liver xanthine dehydrogenase (XDH) is converted into xanthine oxidase (XO), and then XO catalyzes the *de novo* synthesis of uric acid; this conversion leads to the formation of superoxide anions and hydrogen peroxide in parallel to the synthesis of uric acid (Maia et al., 2007). To control this disease, the most commonly considered method involves reducing the production of uric acid by inhibiting XO in the liver. Most commonly used medicinal treatments include allopurinol and febuxostat (Fels and Sundy, 2008). Allopurinol is also an XO inhibitor in present clinical conditions. However, some adverse effects of allopurinol, such as hepatitis, nephropathy, allergic reactions, and bone marrow suppression, have been reported (Fam, 2003). Therefore, there is a need to discover alternatives to xanthine oxidase inhibitors to reduce the risk of hyperuricemia (Nguyen et al., 2004; Wang et al., 2004; Strazzullo and Puig, 2007). Xanthine oxidase is a very important enzyme that has been inhibited by various phenolic compounds (Potapovich and Kostyuk, 2003). Among them, flavonoids are a group of phenolic compounds that are present in many food plants, including aromatic plants such as onions (*Allium cepa* Liliaceae) (Kinoshita et al., 2006). Red onions contain a high level of quercetin, which is a major component of phenolic compounds, along with some flavones, flavanones, isoflavones, catechins, flavanonols, anthocyanidins, and flavonols (Pérez-Gregorio et al., 2010; Arshad et al., 2017). They can help reduce uric acid production by inhibiting the xanthine oxidase enzyme in the liver. Due to the high availability of quercetin in onions, there is a need to recommend its proper amount in the diet of patients with hyperuricemia to reduce their uric acid levels (Miean and Mohamed, 2001; Teyssier et al., 2001). The objective of this study was to use onion powder as a dietary source to reduce uric acid levels in hyperuricemia-induced male rats. Furthermore, feed and water intake, nutrient digestibility, nitrogen balance, body weight, serum biochemical parameters, and hematology in the rats were also studied.

## 2 Materials and methods

### 2.1 Reagents and chemicals

Potassium oxonate (PO) was purchased from Sigma-Aldrich Chemicals. Potassium bromate (KBrO_3_) in the crystalline form was provided by GlaxoSmithKline, Islamabad, Pakistan.

### 2.2 Experimental supplement

Healthy and high-peak-seasoned red onions were purchased from the local market, cut into small pieces, and dried in a hot air oven at 65°C for 24 h. Dried onions were ground into a fine powder using an electrical lab grinder and stored in plastic bags at 4°C until further use (Arslan and Özcan, 2010).

### 2.3 Composition of the diet

The proximate composition of feed and feces was determined using the standard method of AOAC (AOAC, 2006). The diet was isocaloric and isonitrogenous according to the given formulation, as shown in Table 1. In this study, diet was formulated according to the American Institute of Nutrition AIN-93G for the determination of nitrogen metabolism. It contained 3,649.76 kcal/1,000 g energy, as shown in Table 1, including carbohydrates 535.44 g (535.44 × 4 = 2,141.76 kcal), protein 226.97 g (226.97 × 4 = 907.88 kcal), and fats 66.68 g (66.68 × 9 = 600.12 kcal). Therefore, the sum of the total energy from all the nutrients was 2,141.76 + 907.88 + 600.12 = 3,649.76 kcal/kg.

**TABLE 1:** Food ingredients and their estimated nutritive values.

Ingredient (g)	HU		Carbohydrate		Protein		Fat		Total gross energy
Corn starch	154.94		152.94		—		—		—
Maize	111.91		78.44		8.95		4.81		—
Dextrose	152.94		152.94		—		—		—
Corn oil/soybean oil	52.22		—		—		52.22		—
Soybean meal	410.33		125.56		180.54		6.97		—
Canola meal	82.06		25.60		37.48		2.68		—
L-Lysine	1.57		—		—		—		—
DL-Methionine	0.64		—		—		—		—
Tryptophan	0.64		—		—		—		—
Threonine	0.91		—		—		—		—
AIN-93-MX mineral mix	24.5		—		—		—		—
AIN-93-vitamin mix	7		—		—		—		—
tert-Butylhydroquinone (TBHQ)	0.005		—		—		—		—
Total	1,000		535.44		226.97		66.68		—
Total gross energy (kcal/kg)	—		2,141.76		907.88		600.12		3,649.76
CP%	—		—		22.69		—		—
Chemical composition of diets after the addition of various levels of onion powder									
Diet		HU		HOT_1_		HOT_2_		HOT_3_	
Onion powder (g/100 g feed)		—		11.13 (0.75 mg/kg)		14.84 (0.100 mg/kg)		18.61 (0.125 mg/kg)	
Nutritive value feed (%)									
Dry matter		68.25		66		63		68.72	
Crude protein		18.54		18.3		18.4		18.1	
Crude fiber		4.5		5.1		4.42		4.46	
Ether extract		5.8		5.7		5.9		6	
Ash		6		5.8		5.6		5.4	
Moisture		31.75		33		37		31.28	
NFE		33.41		32.1		28.68		34.76	

HU, hyperuricemia; HOT_1_, hyperuricemia onion treatment 1; HOT_2_, hyperuricemia onion treatment 2; HOT_3_, hyperuricemia onion treatment 3; NFE, nitrogen-free extract.

Diet formulation was based on gross energy and ingredients, and nutritional information was sponsored and provided by Refhan Maize and Mukhtar Feeds Ltd. A measure of 100 g of feed was mixed with water and onion powder to make diet pellets. Further proximate analysis was conducted on the resulting feed.

### 2.4 Study design and animals

The study was conducted *in vivo* with the collaboration of the Department of Nutritional Sciences and Department of Physiology at Government College University Faisalabad, Punjab, Pakistan. All procedures were performed according to the guidelines for the management of laboratory animals (Wade, 1978). The study was approved by the Animal Ethical Committee of Government College University Faisalabad, Pakistan (Wide Ref. No. GCUF/ERC/85). Twenty-four adult male Wistar albino rats weighing 180 ± 5 g were housed in the animal laboratory at a temperature of 25°C ± 5°C and humidity of 45%–55%. The rats were randomly divided into four groups based on the diets containing different levels of onion powder: HU (without onion) and HOT_1_, HOT_2_, and HOT_3_ groups treated with doses of onion of 11.13, 14.84, and 18.61 g/100 gm feed corresponding to the concentrations of quercetin of 0.075, 0.100, and 0.125 mg/kg, respectively. Each group comprised six rats, and each group was further divided into two subgroups to ethically control the rats. Each subgroup was fed 100 g diet daily for 3 weeks.

### 2.5 Induction of hyperuricemia in rats

To induce hyperuricemia, a uricase inhibitor called PO (also known as potassium azaorotate, potassium otastat, or oteracil potassium) was used. A dose of 250 mg/kg was prepared in 1 mL of 0.9% saline solution and administered intraperitoneally daily for 14 days. This was done to increase the activity of the XO enzyme and was based on the research conducted by Haidari et al. (2008), Sarvaiya et al. (2015), and Yiying et al. (2016). To further induce the degenerative activity of renal cells and mildly increase the activity of XO, KBrO_3_ was administered orally at 40 mg/kg. The goal was to increase the level of uric acid in serum, according to Kermani et al. (2020).

### 2.6 Evaluation of growth performance and nutrient digestibility

The growth performance parameters and nutrient digestibility were determined according to the following Eqs 1–3 (Nisa et al., 2006; Shi et al., 2015; Naeem et al., 2023). We considered last 7 days as the collection period. Feces were composited, weighed, and stored at −20°C for further analysis. The data were obtained using the following equations:

$$FCR\;\left(\%\right)=\frac{\text{Feed}\;\text{Intake}}{\text{Body}\;\text{Weight}\;\text{Gain}},$$ (1)

$$FER=\frac{Body\;Weight\;Gain}{Feed\;Intake},$$ (2)

$$Digestibility=\frac{Nutrient\;Intake-Nutrient\;in\;feces}{Nutrient\;Intake}\times 100.$$ (3)

### 2.7 Blood collection

At the end of the study, all rats were sacrificed under anesthesia. Blood was collected from the neck area using a surgical blade and preserved in ethylenediaminetetraacetic acid (EDTA) tubes. After centrifuging blood samples at 5,000 rpm, serum was collected and stored at −20°C for further biochemical analysis.

### 2.8 Biochemical analysis

Low-density lipid (LDL) (mg/dL) was calculated using the Friedewald equation (Kaur, 2014). The levels of serum cholesterol (mg/dL), triglycerides (TG) (mg/dL), serum high-density lipid (HDL) (mg/dL), alanine aminotransferase (ALT) (U/L), aspartate aminotransferase (AST) (U/L), serum bilirubin (mg/dL), alkaline phosphatase (ALP) (U/L), total protein (g/dL), creatinine (mg/dL), urea nitrogen, and uric acid (mg/dL) were determined using the Cobas E311 method based on the principle of a spectrophotometer using commercially available kits (Randie Little, 2016). Uric acid kit (CAT No.: UA 121120) (BioMed Diagnostics) was provided by the Department of Nutritional Sciences. The hemoglobin level (Hb) (g/dL); red blood cell (RBC) count (× 10^6^ μL); white blood cell (WBC) count (× 10^3^ µL); monocyte, lymphocyte, neutrophil (%), and platelet count (× 10^5^/µL) were determined using Mindray BC-6200 based on the principle of electrical impedance, flow cytometry, and light scattering (Kulik et al., 2021; Rahim et al., 2023).

### 2.9 Nitrogen balance study

For studying nitrogen balance in rats, urine was collected for 24 h in the following time periods: 6:00 a.m., 8:00 a.m., 10:00 a.m., 12:00 p.m., 2:00 p.m., 4:00 p.m., 6:00 p.m., 8:00 p.m., 10:00 p.m., 12:00 a.m., 2:00 a.m., and 4:00 a.m. This method was adopted after considerable observations in our laboratory. Urine was collected using the palpation and contraction technique, as discussed by Chew and Chua (2003). To perform the procedure, first hold the rat’s tail with the left hand, palpating its lower back near the bladder with the forefinger and middle finger of the other hand and then applying pressure by massaging and rubbing up and down both sides of the lower back near the tail. As the rat began to urinate, the urine was immediately collected in the Eppendorf tubes and stored at −20°C for further analysis. The percentage of nitrogen in feed, urine, and feces was evaluated using the Kjeldahl method (AOAC, 2006). Nitrogen balance involves a 24-h measurement of protein intake and an estimate of nitrogen loss. Nitrogen balance was estimated using Eq. 4:

$$\text{Nitrogen balance}\left(\text{mg}\right)=\left(\text{Nitrigen intake }-\text{ Nitrogen in urine }-\text{ Nitrogen in feces}\right).$$ (4)

### 2.10 Statistics

Statistical software (Statistics 8.1) was used to analyze the data using a complete randomized design. Two-way ANOVA was subjected to analyze weekly feed intake, water intake, and body weight and serum uric acid, whereas other parameters were analyzed by one-way ANOVA, followed by LSD. The level of significance was *p* ≤ 0.05.

### 2.11 Human equivalent dose

In this study, the human equivalent dose of onion is estimated to be 189.95 g with 204 mg of quercetin based on the results, while on dry matter basis, it is 30.62 g with 222 g of quercetin (Eq. 5):

$$Human\;equivalent\;dose\left(\frac{mg}{kg}\right)=Animal\;dose\times Coversion\;factor.$$ (5)

## 3 Results

### 3.1 Feed intake, weight management, and FCR

Feed intake (g) significantly increased (*p* ≤ 0.05) in rats that fed on HOT_1_ diet followed by the HOT_3_ diet, as shown in Table 2. Feed intake was found to be higher in week 1 and week 2 in HOT_1_ and HOT_3_ groups but lower in the HOT_2_ group (191.94 ± 5.9) than that in HU (264.97 ± 8.7). In HOT_3_, there was a significant difference (*p* ≤ 0.05) in feed intake in week 2 compared to week 1 and week 2. Feed intake significantly increased (*p* ≤ 0.05) in week 1 and week 2 in treatment groups, and this might be due to the compensatory growth mechanism. Water intake was significantly increased (*p* ≤ 0.05) from week 1 (158.02 ± 12.49 and 170.83 ± 10.98) to week 3 (184.74 ± 9.62 and 185.82 ± 10.29) in rats that fed on HOT_1_ and HOT_3_ diets compared to the HU diet in week 1 (154.46 ± 8.93) to week 3 (125.63 ± 10.09). According to Table 3, the HOT_2_ treatment group did not show an increasing trend of water intake, and their results are non-significant to HU. Average gain (*p* ≤ 0.05) in body weight (BW) was observed in rats that fed on HU (20.72 ± 0.05 g) and HOT_2_ (22.33 ± 0.05 g) diets. Significant lower (*p* ≤ 0.05) BW (18.01 ± 0.05 g and 18.93 ± 0.05 g) was observed in rats that fed on HOT_3_ and HOT_1_ diets, respectively (Table 4). FCR and FER values were significantly improved in rats that fed on HOT_2_ diet followed by the HOT_1_ diet, which means that rats utilized the feed efficiently and it has positively influenced the body weight compared to HU (Table 5).

**TABLE 2:** Effect of onion on feed intake (gram) in 3 weeks.

Duration of feeding	Treatments			
	HU	HOT_1_	HOT_2_	HOT_3_
Week 1	264.97 ± 8.7^a^	245.26 ± 6.68^a^	214.67 ± 7.47^a^	235.94 ± 6.36^b^
Week 2	235.27 ± 7.29^b^	249.52 ± 7.98^a^	191.94 ± 5.9^b^	263.03 ± 7.34^a^
Week 3	234.44 ± 6.61^b^	243.85 ± 6.61^a^	205.47 ± 8.22^a^	233.08 ± 5.71^b^
Total	734.78 ± 0.1^b^	738.67 ± 0.14^a^	612.11 ± 0.09^d^	732.12 ± 0.1^c^

HU, hyperuricemia; HOT_1_, hyperuricemia onion treatment 1; HOT_2_, hyperuricemia onion treatment 2; HOT_3_, hyperuricemia onion treatment 3.

**TABLE 3:** Effect of onion on water intake (mL) in 3 weeks.

Duration of feeding	Treatments			
	HU	HOT_1_	HOT_2_	HOT_3_
Week 1	154.46 ± 8.93^a^	158.02 ± 12.49^b^	166.31 ± 12.52^a^	170.83 ± 10.98^a^
Week 2	124.69 ± 8.60^b^	178.97 ± 9.51^a^	117.62 ± 12.79^b^	171.26 ± 12.99^a^
Week 3	125.63 ± 10.09^b^	184.74 ± 9.62^a^	125.72 ± 14.04^b^	185.82 ± 10.29^a^
Total	404.80 ± 0.12^d^	521.77 ± 0.13^b^	409.69 ± 0.13^c^	527.91 ± 0.075^a^

HU, hyperuricemia; HOT_1_, hyperuricemia onion treatment 1; HOT_2_, hyperuricemia onion treatment 2; HOT_3_, hyperuricemia onion treatment 3.

**TABLE 4:** Effect of onion on body weight (grams).

Group	HU	HOT_1_	HOT_2_	HOT_3_
Initial body weight (before treatment)	179.32 ± 0.23^c^	180.30 ± 0.24^b^	180.81 ± 0.15^a^	180.90 ± 0.08^a^
Final body weight (after treatment)	200.04 ± 0.23^b^	199.23 ± 0.26^c^	203.14 ± 0.21^a^	198.92 ± 0.14^c^
Average body weight gain	20.72 ± 0.05^b^	18.93 ± 0.05^c^	22.33 ± 0.05^a^	18.01 ± 0.05^d^

HU, hyperuricemia; HOT_1_, hyperuricemia onion treatment 1; HOT_2_, hyperuricemia onion treatment 2; HOT_3_, hyperuricemia onion treatment 3.

**TABLE 5:** Effect of onion on feed conversion and feed efficiency ratios (%).

Group	HU	HOT_1_	HOT_2_	HOT_3_
Total feed intake in 3 weeks (g)	734.78 ± 0.1^b^	738.67 ± 0.14^a^	612.11 ± 0.09^d^	732.12 ± 0.1^c^
Total weight gain in 3 weeks (g)	20.72 ± 0.05^b^	18.93 ± 0.05^c^	22.33 ± 0.05^a^	18.01 ± 0.05^d^
FCR (%)	35.528 ± 0.18^c^	39.085 ± 0.17^b^	27.458 ± 0.14^d^	40.683 ± 0.12^a^
FER	0.0300 ± 0.009^a^	0.0278 ± 0.008^a^	0.0388 ± 0.009^a^	0.0263 ± 0.008^a^

FCR, feed conversion ratio; FER, feed efficiency ratio; HU, hyperuricemia; HOT_1_, hyperuricemia onion treatment 1; HOT_2_, hyperuricemia onion treatment 2; HOT_3_, hyperuricemia onion treatment 3.

### 3.2 Nutrient intake (gm)

Higher values of DM intake (22.51 ± 0.14 and 22.47 ± 0.20) were observed in rats that fed on HOT_3_ and HOT_2_ diets, and lower DM intake was observed in rats that fed on HOT_1_ (17.60 ± 0.14) than HU (22.05 ± 0.11). Higher CP intake (6.27 ± 0.20) was observed in rats that fed on HOT_1_ diet than HOT_2_ (5.18 ± 0.17), HOT_3_ (5.95 ± 0.14), and HU (6.08 ± 0.23). A similar trend followed in CF intake, and higher (1.78 ± 0.10) fiber intake was observed in rats that fed on HOT_1_ diet than in the other treatment groups. Ether extract (EE) intake was found to be higher (2.00 ± 0.13 and 1.95 ± 0.07) in rats that fed on HOT_3_ and HOT_1_ diets than in HOT_2_ (1.69 ± 0.12) and HU (1.91 ± 0.14). Ash intake was found to be higher (2.02 ± 0.10) in rats that fed on HOT_1_ diet than in HOT_2_ (1.60 ± 0.14), HOT_3_ (1.81 ± 0.14), and HU (1.97 ± 0.07) diets (Table 6).

**TABLE 6:** Effect of onion on nutrient intake (grams).

Group	HU	HOT_1_	HOT_2_	HOT_3_
Dry matter	22.05 ± 0.11^b^	22.47 ± 0.20^a^	17.60 ± 0.14^c^	22.51 ± 0.14^a^
Crude protein	6.08 ± 0.23^ab^	6.27 ± 0.20^a^	5.18 ± 0.17^c^	5.95 ± 0.14^b^
Crude fiber	1.51 ± 0.13^b^	1.78 ± 0.10^a^	1.25 ± 0.18^c^	1.49 ± 0.12^b^
Ether extract	1.91 ± 0.14^a^	1.95 ± 0.07^a^	1.69 ± 0.12^b^	2.00 ± 0.13^a^
Ash	1.97 ± 0.07^ab^	2.02 ± 0.10^a^	1.60 ± 0.14^c^	1.81 ± 0.14^b^

HU, hyperuricemia; HOT_1_, hyperuricemia onion treatment 1; HOT_2_, hyperuricemia onion treatment 2; HOT_3_, hyperuricemia onion treatment 3.

### 3.3 Nutrient digestibility (%)

Significant DM digestibility was found to be higher (*p* ≤ 0.05) in rats that fed on HOT_3_ (93.47 ± 0.35) and HU (92.51 ± 0.27) diets than in HOT_2_ (90.44 ± 0.30) and HOT_1_ (90.81 ± 0.12) diets. Similar trend in the digestibility of CP, ash, and nitrogen-free extract (NFE) was observed to be higher (*p* ≤ 0.05) in HOT_3_ and HU diets than the other groups. Higher (91.15% ± 0.29%) and lower (89.08 ± 0.29) CF digestibility was observed in HOT_3_ and HOT_1_ diets compared to the HOT_2_ (88.60 ± 0.29) and HU (89.14 ± 0.37). EE digestibility was significantly improved (*p* ≤ 0.05) in rats that fed on HOT_3_ (90.56% ± 0.23%), HOT_1_ (89.51 ± 0.32), and HOT_2_ (89.44 ± 0.21) diets compared to HU (89.32 ± 0.23) (Table 7).

**TABLE 7:** Effect of onion on nutrient digestibility (%).

Nutrient	HU	HOT_1_	HOT_2_	HOT_3_
Dry matter	92.51 ± 0.27^b^	90.81 ± 0.12^c^	90.44 ± 0.30^c^	93.47 ± 0.35^a^
Crude protein	91.30 ± 0.27^b^	91.31 ± 0.18^b^	91.19 ± 0.26^b^	92.55 ± 0.25^a^
Crude fiber	89.14 ± 0.37^b^	89.08 ± 0.29^b^	88.60 ± 0.29^c^	91.15 ± 0.29^a^
Ether extract	89.32 ± 0.23^b^	89.51 ± 0.32^b^	89.44 ± 0.21^b^	90.56 ± 0.23^a^
Ash	89.65 ± 0.19^b^	89.43 ± 0.31^b^	88.88 ± 0.24^c^	91.23 ± 0.27^a^
NFE	94.90 ± 0.20^a^	91.80 ± 0.17^b^	91.13 ± 0.35^c^	94.98 ± 0.14^a^

HU, hyperuricemia; HOT_1_, hyperuricemia onion treatment 1; HOT_2_, hyperuricemia onion treatment 2; HOT_3_, hyperuricemia onion treatment 3; NFE, nitrogen free extract.

### 3.4 Uric acid and renal and liver functions

Serum uric acid was significantly induced (*p* ≤ 0.05) in rats, and higher uric acid levels were found at day 14 in the treatment groups. The latest changes are discussed in this section. Uric acid was successfully induced (*p* ≤ 0.05) in all the treatment groups, and it was found to be higher (4.80 ± 0.12 mg/dL and 4.20 ± 0.36 mg/dL) in rats that fed on the HOT_3_ and HOT_1_ diets on day 14, respectively. The treatment period started from day 14 to day 28. The serum uric acid levels significantly decreased (3.01 ± 0.54 mg/dL and 3.21 ± 0.11 mg/dL) in rats that fed on HOT_1_ and HOT_3_ diets, respectively. To explore the further efficacy of dried onion, the treatment period was extended until day 35, and the levels of serum uric acid were significantly decreased (*p* ≤ 0.05) (1.53 ± 0.08 mg/dL and 1.81 ± 0.07 mg/dL) in rats that fed on HOT_2_ and HOT_3_ diets, respectively, compared to HU (4.31 ± 0.03 mg/dL), as shown in (Table 8). Significantly lower levels of creatinine (0.70 ± 0.2 mg/dL and 0.44 ± 0.05 mg/dL) were observed in rats that fed on HOT_2_ and HOT_3_ diets compared to HU. Serum blood urea nitrogen (BUN) was found to be lower (32.49 ± 4.69 mg/dL and 33.64 ± 2.12 mg/dL) in rats that fed on HOT_3_ and HOT_2_ diets than the rats that fed on HOT_1_ (45.66 ± 4.16 mg/dL) and HU (35.33 ± 5.50 mg/dL) diets (Table 9). Serum ALT, AST, and ALP (U/L) were significantly (*p* ≤ 0.05) decreased in rats that fed on onion diets. A decreasing trend (*p* ≤ 0.05) in bilirubin levels (1.49 ± 0.14 mg/dL and 1.74 ± 0.12 mg/dL) was observed in rats that fed on HOT_3_ and HOT_2_ diets than HOT_1_ (3.94 ± 0.21 mg/dL) and HU (3.76 ± 0.55 mg/dL) diets (Table 10).

**TABLE 8:** Effect of onion on the uric acid level (mg/dL).

Day	Treatments			
	HU	HOT_1_	HOT_2_	HOT_3_
Day 0	0.95 ± 0.03^d^	0.82 ± 0.065^c^	0.82 ± 0.08^d^	0.90 ± 0.05^d^
Day 14	4.05 ± 0.03^c^	4.20 ± 0.36^a^	4.13 ± 0.04^a^	4.80 ± 0.12^a^
Day 28	4.20 ± 0.06^b^	3.01 ± 0.54^b^	3.52 ± 0.06^b^	3.21 ± 0.11^b^
Day 35	4.31 ± 0.03^a^	2.78 ± 0.58^b^	1.53 ± 0.08^c^	1.81 ± 0.07^c^

HU, hyperuricemia; HOT_1_, hyperuricemia onion treatment 1; HOT_2_, hyperuricemia onion treatment 2; HOT_3_, hyperuricemia onion treatment 3.

**TABLE 9:** Effect of onion on renal function (mg/dL).

Treatment	HU	HOT_1_	HOT_2_	HOT_3_
Creatinine	1.43 ± 0.15^a^	0.73 ± 0.15^bc^	0.70 ± 0.2^bc^	0.44 ± 0.05^d^
BUN	35.33 ± 5.50^b^	45.66 ± 4.16^a^	33.64 ± 2.12^b^	32.49 ± 4.69^b^

HU, hyperuricemia; HOT_1_, hyperuricemia onion treatment 1; HOT_2_, hyperuricemia onion treatment 2; HOT_3_, hyperuricemia onion treatment 3; BUN, blood urea nitrogen.

**TABLE 10:** Effect of onion on liver function.

Group	HU	HOT_1_	HOT_2_	HOT_3_
ALT (U/L)	238.67 ± 8.02^a^	119.33 ± 12.05^c^	54.463 ± 1.56^e^	84.62 ± 3.21^d^
AST (U/L)	352.77 ± 10.52^a^	125.64 ± 5.75^bc^	82.72 ± 10.53^c^	104.71 ± 14.20^bc^
Total bilirubin (mg/dL)	3.76 ± 0.55^b^	3.94 ± 0.21^b^	1.49 ± 0.14^c^	1.74 ± 0.12^c^
ALP (U/L)	385.33 ± 10.06^a^	241.33 ± 20.55^d^	160.00 ± 20.29^e^	164.33 ± 19.00^e^

ALT, alanine aminotransferase; AST, aspartate aminotransferase; ALP, alkaline phosphatase; HU, hyperuricemia; HOT_1_, hyperuricemia onion treatment 1; HOT_2_, hyperuricemia onion treatment 2; HOT_3_, hyperuricemia onion treatment 3.

### 3.5 Serum total protein and lipid profile

Significantly improved serum total protein levels (6.85 ± 0.07 mg/dL and 6.86 ± 0.10 mg/dL) were observed in rats that fed on HOT_2_ and HOT_3_ diets compared to HOT_1_ (6.76 ± 0.06 mg/dL) and HU (6.78 ± 0.16 mg/dL) diets (Table 11). In serum total cholesterol, LDL was significantly reduced (*p* ≤ 0.05) in rats that fed on onion-enriched diets compared to HU. The level of HDL did not increase (*p* ≤ 0.05) in rats that fed on HOT_3_ (18.75 ± 2.18 mg/dL) and HOT_2_ (24.42 ± 2.63 mg/dL), but HOT_1_ (30.10 ± 2.11 mg/dL) had not differed significantly (*p* ≥ 0.05) compared to HU (30.70 ± 2.52 mg/dL) (Table 12).

**TABLE 11:** Effect of onion on serum total protein.

Group	HU	HOT_1_	HOT_2_	HOT_3_
Total protein (g/dL)	6.78 ± 0.16^b^	6.76 ± 0.06^b^	6.85 ± 0.07^ab^	6.86 ± 0.10^ab^

HU, hyperuricemia; HOT_1_, hyperuricemia onion treatment 1; HOT_2_, hyperuricemia onion treatment 2; HOT_3_, hyperuricemia onion treatment 3.

**TABLE 12:** Effect of onion on the lipid profile (mg/dL).

Treatment	HU	HOT_1_	HOT_2_	HOT_3_
Cholesterol	136.33 ± 5.68^a^	94.08 ± 4.34^cd^	87.69 ± 1.55^d^	134.67 ± 14.57^a^
HDL	30.70 ± 2.52^a^	30.10 ± 2.11^a^	24.42 ± 2.63^b^	18.75 ± 2.18^c^
Triglycerides	165.00 ± 9.53^c^	82.66 ± 19.13^e^	134.00 ± 8.18^d^	187.67 ± 16.86^bc^
LDL	72.63 ± 9.77^a^	47.44 ± 4.38^bc^	36.47 ± 2.96^bc^	78.38 ± 10.90^a^

HDL, high-density lipid; LDL, low-density lipids; HU, hyperuricemia; HOT_1_, hyperuricemia onion treatment 1; HOT_2_, hyperuricemia onion treatment 2; HOT_3_, hyperuricemia onion treatment 3.

### 3.6 Effects of onions on body immunity and hematology

Higher WBC count was observed in rats that fed on onion-containing diets; however, HOT_1_ had shown significantly higher (15.89 ± 0.27 × 10^3^ µL) WBC count than other treatment groups. Significant improvement (*p* ≤ 0.05) was observed in neutrophil and lymphocyte percentages among onion-treated groups compared to HU. Monocytes were considerably found to be higher in rats that fed on HOT_1_ (8.70% ± 0.2%) and HOT_2_ (8.63% ± 0.20%) diets than HU. The RBC count was significantly found to be higher (*p* ≤ 0.05) in rats that fed on HOT_2_ (7.21 ± 0.22 × 10^6^µL) than those in the other groups. Higher (13.32 ± 0.06 g/dL) and lower (13.23 ± 0.25 g/dL) Hb levels were observed in rats that fed on onion-treated diets compared to the other diets. Similarly, in platelet count, onion-enriched diets had shown an improvement compared to HU. It was significantly improved (*p* ≤ 0.05) in onion-supplemented diets, and a higher value (682.33 ± 30.27 × 10^5^/µL) of platelet count was observed in rats that fed on HOT_1_ diet compared to other treatment groups (Table 13).

**TABLE 13:** Effect of onion on body immunity and hematology.

Group	HU	HOT_1_	HOT_2_	HOT_3_
White blood cell count (× 10^3^ µL)	14.12 ± 0.22^e^	15.89 ± 0.27^b^	15.41 ± 0.22^cd^	15.82 ± 0.15^bc^
Neutrophils %	0.76 ± 0.15^d^	4.50 ± 0.3^a^	4.31 ± 0.19^a^	4.17 ± 0.16^a^
Lymphocytes %	90.067 ± 1.00^b^	79.41 ± 1.68^c^	102.52 ± 4.09^a^	83.40 ± 0.92^c^
Monocytes %	8.36 ± 0.25^ab^	8.70 ± 0.2^a^	8.63 ± 0.20^ab^	8.26 ± 0.30^b^
RBC (× 10^6^ μL)	6.99 ± 0.07^abc^	6.35 ± 0.09^e^	7.21 ± 0.22^a^	6.82 ± 0.10^c^
Hb (g/dL)	12.92 ± 0.13^d^	13.32 ± 0.06^bcd^	13.23 ± 0.25^cd^	13.31 ± 0.44^bcd^
Platelet (PLT) (× 10^5^/µL)	574.33 ± 12.22^e^	682.33 ± 30.27^c^	642.00 ± 17.08^d^	665.67 ± 8.50^cd^

RBC, red blood cell; HB, hemoglobin; HU, hyperuricemia; HOT_1_, hyperuricemia onion treatment 1; HOT_2_, hyperuricemia onion treatment 2; HOT_3_, hyperuricemia onion treatment 3.

### 3.7 Effects of onion on nitrogen balance

Positive nitrogen balance (0.521 ± 0.01 mg/day) was observed (*p* ≤ 0.05) in rats that fed on the HOT_1_ diet, but HOT_2_ diet did not show any improvement in nitrogen balance. HOT_3_ (0.480 ± 0.006 mg/day) had shown non-significant (*p* ≥ 0.05) results in the nitrogen balance study compared to HU (0.484 ± 0.001 mg/day) (Table 14).

**TABLE 14:** Effect of onion on nitrogen balance.

Parameters (mg/day)	HU	HOT_1_	HOT_2_	HOT_3_
N-intake	0.961 ± 0.01^b^	0.99 ± 0.004^a^	0.82 ± 0.01^c^	0.94 ± 0.01^b^
Urinary-N	0.140 ± 0.007^a^	0.134 ± 0.009^ab^	0.138 ± 0.008^a^	0.122 ± 0.009^b^
Fecal-N	0.336 ± 0.005^a^	0.340 ± 0.01^a^	0.334 ± 0.007^a^	0.345 ± 0.006^a^
N-balance	0.484 ± 0.001^b^	0.521 ± 0.01^a^	0.353 ± 0.004^c^	0.480 ± 0.006^b^

HU, hyperuricemia; HOT_1_, hyperuricemia onion treatment 1; HOT_2_, hyperuricemia onion treatment 2; HOT_3_, hyperuricemia onion treatment 3.

## 4 Discussion

Hyperuricemia is a condition where uric acid levels in the body are imbalanced, leading to joint pain and kidney problems. Drugs like allopurinol are used to treat hyperuricemia , but natural medicinal plants are being researched as a potential cure. The groups fed with onions showed a significant improvement in their feed intake (g) compared to the positive control group (HU). After the adaptation period, the rats’ feed intake improved due to the addition of onions in their diet, which attracted more attention. According to Table 2, feed intake was lowered during the third week due to the compensatory growth mechanism. However, feed intake was improved until the third week in HOT_2_. Onions contain fructooligosaccharide and inulin, which are fibers that aid in digestion and absorption by increasing the activity of healthy bacteria in the gut. Additionally, onions contain essential vitamins and minerals, such as vitamin C, vitamin B6, potassium, and folate, which contribute to animal health and growth performance (Fernández-Jalao et al., 2021; Rahim et al., 2021). Onions also enhance feed intake by reducing inflammation in gastrointestinal lining (Sangpreecha et al., 2023). Similar studies have shown that feed intake is improved in broiler chicks when fed on 100 mg of onion powder in water (Aji et al., 2011). The antifungal, anti-inflammatory, and antibacterial properties of onions also improve digestion and absorption by halting the activity of pathogenic microbes and enhancing the activity of commensal microbes (Farahani et al., 2015). However, Al-Homidan (2005) observed that 2% onion powder did not impact the feed intake, and the addition of 6% onion to the diet decreased feed intake in broilers. During the experimental period, water intake was also observed, which is influenced by the presence of fiber and protein digestion and absorption activities in the body. Weekly water intake (mL) in HU was significantly decreased as a result of degenerative changes produced by PO and potassium bromate (Table 3). Water intake was observed to be higher in rats fed with onion diets, which may be associated with their higher protein and fiber intake. Similar results of high water intake were also observed by Ulger and Cakiroglu (2020), who provided 5% lyophilized and furnaced onion to diabetic rats.

In the experimental period, higher BW was observed in induced rats than other studies where BW was reduced by the administration of PO and potassium bromate. It had been observed that despite the adverse effects of PO and potassium bromate (Ajarem et al., 2016; Al-Masri, 2016; Park et al., 2018), BW was increased within the treatment groups, and this might be due to a high protein diet and high feed intake. As shown in Table 4, fluctuation in BW observed in rats that fed on onion-enriched diets may be due to the presence of quercetin, rutin, sulfur, and phenolic compounds, and these compounds have antioxidant and anti-inflammatory effects on the body (Slimestad et al., 2007). Al-Masri, (2016) proposed that BW decreased in rats fed with 2% PO for 6 weeks. Elberry et al. (2014) explored the antioxidant and antimicrobial effects of onions that influence gain in body weight. Similarly, Goodarzi et al. (2014) also found BW gain in broiler chicks that fed on diet containing 3% onion bulbs. Hassan et al. (2018) also found a positive impact on BW in female Ross-308 broiler chicks that were provided rutin at various levels. Rutin is a subtype of quercetin and has antioxidant and anti-inflammatory properties.

Feed conversion and feed efficiency ratios are the indications of growth performance parameters, feed conversion ratio is feed intake per body weight gain, and feed efficiency is the body weight gain per feed intake. An improvement in values of FCR and FER were observed among the rats fed with onion diets, and this positive influence was associated with the concentration of protein available from the diet. According to Table 5, HOT_2_ showed greater improvement in FCR (%) and FER than the other treatment groups. This could be because onions have antibacterial properties that prevent harmful pathogens from multiplying while also widening the intestinal surface to aid in amino acid absorption. Goodarzi et al. (2014) and Bedford (2000) found that herbs and phytogenic compounds were more effective at controlling harmful bacteria in chick guts than antibiotics. Onions contain other beneficial compounds, such as polyphenols, terpenoids, polypeptides, lectins, alkalis, and essential oils, which also positively impact digestion, leading to improved FCR and FER values. However, Rohn et al. (2002) and Swieca et al. (2013) found that flavonoids can interact with nutrients and reduce the activity of digestive enzymes, resulting in no improvement in FCR and FER values.

Nutrient intake is a critical factor that both humans and animals must prioritize. It is essential for fulfilling energy needs, supporting growth and maintenance, boosting immunity, promoting reproduction, and preventing diseases. Therefore, consuming necessary nutrients is crucial for various physiological functions. The rats that fed on HOT_1_ and HOT_3_ diets showed a significantly higher intake of DM and EE (g) than those in the HU group, with HOT_1_ demonstrating a higher intake of CP, CF, and ash, as shown in Table 6. The difference in nutrient intake among the treatment groups could be attributed to high feed intake, nutrient digestibility, anti-quality compounds in onions, and onion-to-feed ratio. Aditya et al. (2017) attribute the positive impact of onion and quercetin on nutrient intake to their antioxidant, anti-inflammatory, and prebiotic properties.

Apparent nutrient digestibility describes the status of ingested nutrients in the body. It showed the difference between the amount of feed consumption, feed utilization, and nutrients present in the feces. It is shown in general terms, including DM, CP, EE, NFE (carbohydrates), and ash digestibilities. It also revealed the digestibility of energy and nitrogen in the diet and feces. In the present trial, CP and EE digestibility was found to be higher in HOT_1_ and HOT_3_ groups than HOT_2_ and HU groups. Significantly higher DM, CF, ash, and NFE digestibility was observed in rats that fed on HOT_3_ diet compared with HOT_1_, HOT_2_, and HU diets. A variation in digestibility among the groups may occur due to the higher feed and nutrient intake. HOT_2_ had shown lower CP and EE digestibility due to the less feed intake and associated with the compensatory growth mechanism, as previously discussed in feed intake in Table 7. Onions contain nutrients such as protein, fat, fiber, vitamins, and minerals that facilitate digestion and absorption (Mitra et al., 2012). Quercetin acts as a growth promoter and improves the digestion system functionality by enhancing the activity of commensal microbes (Saeed et al., 2017). In the same manner, onions improve the activity of digestive enzymes and plays a role as a prebiotic due to soluble fiber contents like inulin and fructooligosaccharides (Gibson, 1998; Binaii et al., 2014; Safari et al., 2014; Mousavi et al., 2016). In contrast to our findings, another study reported the beneficial effects of onions, including increasing the nutrient digestibility and promoting the growth and development in the body by creating a balance in the gut microbiota. In the colon, beneficial microbes significantly enhance the fermentation of fiber content and produce short-chain fatty acids that comply with the energy needs of the body (Nakano, 2007; Ye et al., 2011).

Hyperuricemia is linked to various medical disorders in the body, such as joint pain, renal stones, and inflammation. During the breakdown of purine and pyrimidines, XO) plays an important role in catalyzing the production of uric acid by the conversion of hypoxanthine to xanthine and uric acid. Potassium oxonate (a uricase inhibitor) enhances XO activity and synthesizes uric acid in the body. This study presented that the levels of serum uric acid were significantly lower in rats that fed on HOT_2_ and HOT_3_ diets, as shown in Table 8. Onions have a higher capacity to reduce serum uric acid levels than quercetin aglycone alone. This could be due to flavonoids in onions having higher intestinal absorption and bioavailability than quercetin (Hertog et al., 1992; Haidari et al., 2008). In the present investigation, onion as a whole food having various concentrations of quercetin was explored for its potent effect to reduce uric acid levels. Contrary to the statement made previously, Zhu et al. (2004) conducted an experiment that administered *Biota orientalis* (BO) extract, quercetin, and rutin at a 100 mg/kg level to hyperuricemia-induced mice. Both substances demonstrated potential effects in reducing serum uric acid levels, with BO extract surpassing the positive control in effectiveness. The result suggests that quercetin and rutin have the ability to bind to XO and regulate serum uric acid levels. Quercetin also regulates the organic anion transporters (mOAT1) responsible for the absorption and excretion of uric acid in the kidney’s proximal tubules. In comparison to the previous study, quercetin was found to reduce uric acid levels by increasing the expression of mOAT1 in fructose-induced rats (Hu et al., 2009; Hu et al., 2012). The expression of mOAT1 facilitates the removal of uric acid from the body (Hong et al., 2007). However, it is worth noting that the intake of flavonoids in food and supplements may sometimes interfere with the expression of mOAT (Wong et al., 2011). This may form a link among the onion-treated groups, which did not show a higher effect on serum uric acid levels. In another *in vitro* study, red onion polyphenols hold more promising antioxidant and XO inhibition activities. According to the previous results, polyphenols in red onions had high antioxidant activity, and they restricted the XO inhibition activity up to IC_50_ 17.36 mg/mL (Ouyang et al., 2018). Another study conducted by Mo et al. (2007) evaluated the hypouricemic effects of 15 dietary flavonoids, namely, quercetin, morin, myricetin, kaempferol, icariin, apigenin, luteolin, baicalin, silibinin, naringenin, formononetin, genistein, puerarin, daidzin, and naringin dihydrochalcone. Hyperuricemia was induced with intraperitoneal injection of PO, and after 1 h, tested flavonoids and allopurinol were administered orally for 3 days. Quercetin, myricetin, morin, puerarin, and kaempferol at the level of 50 and 100 mg/kg had shown a decreasing trend in the liver XO activity and also elicited a declining effect in serum uric acid levels.

Reports indicate that biochemical and morphological changes occur after the adverse effects of PO and potassium bromate induction (Ahmed et al., 2016; Vijeesh et al., 2022). Induction with PO and potassium bromate may have adversely affected the renal function by changing renal parameters, and it adversely affects the renal parenchyma and decreases the glomerular filtration rate (Khan et al., 2003). If the kidney glomerular filtration rate decreases, then it means kidneys are not in a position to filter the waste materials from the body resulting in many complications. An increase in creatinine and BUN truly shows the decrease in the glomerular filtration rate resulting from morphological and structural changes in the renal tubules (Gowda et al., 2010). According to Table 9, our findings showed that the renal parameters were attenuated, which can be attributed to onion’s anti-inflammatory and antioxidant activities. The increased serum uric acid level and reactive oxygen species (ROS) production may have resulted from the degradation of purine and pyrimidines and an increase in the activity of XO. Quercetin in the onion may have the capacity to bind to XO and decrease the synthesis of uric acid in the body (Mohamed and Saddek, 2019). Onions enhanced the antioxidant status and restored the renal parameters that had been approved in the study conducted by Rahmat et al. (2019), who provided 5 g/kg/day onion to the hyperuricemic rats. Quercetin lowers the harmful effects by scavenging free radicals and inhibiting lipid peroxidation and XO enzyme activity (Frankel et al., 1993). Flavonoids are found in various fruits and vegetables which hold antioxidant activity, and they are widely distributed into various groups and categories according to their chemical structures and therapeutic values. Quercetin significantly attenuated the renal morphology and function by increasing the glomerular filtration rate. It might be due to the anti-inflammatory activity of quercetin (Gomes et al., 2014; Tang et al., 2020).

In this study, HOT_2_ and HOT_3_ significantly reduced the levels of ALT, AST, ALP, and total bilirubin, as shown in Table 10. Onion contains numerous functional compounds, such as quercetin and sulfur. It attenuates the liver enzymes by protecting the liver cell from oxidative stress and inflammation. Quercetin in the onion scavenges free radicals and protects the cells from lipid peroxidation. Lipid peroxidation is the chain of reactions that causes oxidative damage to the cell layer of the liver. Most often, the phospholipid layer of the cell gets damaged due to lipid peroxidation. When onion and quercetin are added to the diet, it lowers the lipid levels in the liver along with improvement in BW, which results in a decrease in liver enzymes. Onion contains fiber, sulfur compounds, and flavonoids that have shown good results compared to quercetin alone (Lee et al., 2008). There are various health benefits of onions that have been reported, including antibacterial, antifungal, anti-atherosclerotic, hypolipidemic, antihypertensive, hypoglycemic, anti-inflammatory, and antioxidant properties. These therapeutic effects have directly or indirectly improved liver morphology and functions (Ozougwu et al., 2008; Eyo et al., 2011; Ozougwu, 2011). From the diseased liver or fatty liver, enzymes ooze out from the cell into the serum. Kuo et al. (2012) induced hyperuricemia in rats with an intraperitoneal injection of potassium oxonate at 280 mg/kg for 1 week. *Hibiscus sabdariffa* L. provided at the level of 1%, 2%, and 5% in the diet significantly attenuated the levels of uric acid, ALT, and AST. Both are important enzymes that are present in the cytoplasm and mitochondria of the liver (Amacher, 1998). Ozer et al. (2008) also investigated the mechanism of liver damage and injury. Bilirubin is the end product of RBCs and is mostly found in the liver, bile, intestine, and spleen, whereas ALP and 𝛾-GT are found in the cell membrane (Ramaiah, 2007). Our study was correlated with the study conducted by de David et al. (2011) and Mishra et al. (2013), who found quercetin’s capacity to reverse the levels of bilirubin, ALT, AST, ALP, and 𝛾-GT attributed to its hepatoprotective effect. In another study, quercetin at 20 mg/kg provided to the rats had protected the rats from melphalan (MPLN)-induced toxicity. It may be attributed to anti-inflammatory and antioxidant activity of quercetin (Olayinka et al., 2014). Table 11 shows that with the improvement in the functioning of the liver, serum total protein also improved among the rats that fed on onion-treated diets (Karakilcik et al., 2004).

The high levels of uric acid induce gluconeogenesis in the liver, stimulating adenosine monophosphate dehydrogenase, lowering the activity of adenosine monophosphate kinase, and causing a disturbance in lipid metabolism that can lead to hepatic lipogenesis (Cicerchi et al., 2014; Lanaspa et al., 2015). Table 12 shows that rats that fed on HOT_1_ and HOT_2_ diets experienced a greater decrease in lipid levels than those fed on HOT_3_ diet due to the higher feed intake and onion-to-feed ratio in HOT_1_ and HOT_2_ diets. Onions contain various sulfur compounds, such as s-methyl cysteine, that can increase HDL levels and lower LDL levels, triglycerides, and HMG CoA reductase activity (Thomas et al., 2015). Onions significantly reduced lipid levels in streptozotocin-induced diabetic rats due to the presence of allyl propyl disulfide (Ozougwu, 2011). However, rats that fed on high-cholesterol diet with 10% onion powder did not show any changes in total cholesterol, LDL, or triglyceride levels (González-Peña et al., 2014). Quercetin restricts the working of lipase enzymes and lowers *de novo* lipid synthesis in the liver, as found in the study conducted by Gnoni et al. (2009). Rutin (quercetin-3-rutinoside), a subtype of quercetin found in various plants and fruits, also reduces lipid levels by restricting the activity of the PPAR enzyme in the liver, which is important for lipid synthesis (Ahmadipour et al., 2015). In rabbits that fed on a high-fat diet, quercetin had a positive effect on the lipid profile (Juzwiak et al., 2005).

The experimental studies that have been accomplished to study the organism status and conditions are adopted according to the laboratory protocols. Various biochemical and hematological tests are performed to explore the status of disease, and new therapeutic strategies are developed and validated using the scientific studies. The present research has been conducted to explore the negative effect of PO and potassium bromate on the hematological parameters and to find out the effect of onion powder on the hematological values in hyperuricemia-induced albino rats. According to the study results, the levels of WBCs, RBCs, and Hb were significantly improved in rats that fed on the HOT_1_, HOT_2_, and HOT_3_ diets compared to HU, as shown in Table 13. This may be attributed to antioxidant, anti-inflammatory, and antibacterial activities of onions. Some other contributing factors include higher CP intake and digestibility as well as an increase in the synthesis of serum protein in the liver. The most important function of the liver is to maintain hemopoiesis and the formation of coagulation proteins. Disturbance in the liver functions and morphology might have led to the disturbance in the hematological parameters. Higher intake of protein may improve the immunological response and hematological values in the body. Serum parameters such as RBC, WBC, and platelet count have been indicated as essential biomarkers in various clinical conditions. Due to the different morphological structures and shape of RBCs, they play an important role in transporting oxygen and nutrients to all parts of the body (Barbalato and Pillarisetty, 2020). Ufele et al. (2008) found positive variations in RBC levels in mice that fed on plant-based protein (soybean and corn meal). Similarly, higher RBCs were found due to the bioavailability of animal protein than plant-based protein (Aguirre et al., 1959). Contrary to this study, Kim et al. (2014) reported a decrease in WBC count upon providing a high level of plant-based protein. WBC count has been known for their active role in immunity, including innate and humoral immune response. They are also called leukocytes. They encounter foreign substances and protect the body from harmful exposure to infectious substances (Ashton, 2007; Tigner et al., 2021). Platelet count play an important role in maintaining the level of blood through the activation of blood clotting factors in various type of vascular injury (Fountain and Lappin, 2020). The diet composition contains more protein content from the soybean meal, and its bioavailability is 70% compared to 86% animal protein. Animal proteins such as those present in meat, poultry, fish, and dairy products are more abundant in the heme-iron, which is readily absorbed by the body compared with the plant-based protein. Plant protein contains non-heme-iron, which has a less absorption level, such as legumes, fortified cereals, and leafy greens. However, it has a positive effect on hematological values. The levels of WBC and RBC count were shown to be improved in the study conducted by Akrami et al. (2013), who provided 1% onion powder to the fish. Quercetin in the onion has anti-inflammatory and immune-boosting activity. Feeding onion to grass carp resulted in an improvement in the Hb level, WBC count, and RBC count, and it also revealed onion’s immunomodulating and antibacterial activity. Lymphocyte and neutrophil levels were found to be non-significant in onion-treated groups but found to be higher compared with HU. Monocytes also significantly improved in the rats that fed on onion diets (Gholipour Kanani et al., 2014). Keskin et al. (2016) and Aguirre et al. (2011) reported that RBC count was increased by the supplementation of quercetin. Quercetin in the onion reduces the inflammation in the body and protects RBCs from free radicals by increasing their oxygen-carrying capacity. Nazima et al. (2016) also highlighted the positive effect of quercetin on hematological values and explored its protective effect on erythrocyte cell membranes from free radical exposure. Similarly, positive effects on hematological parameters had been studied in rats that fed on diets treated with garlic and onion powders (Kalyankar et al., 2013). In another study, quercetin injected intraperitoneally at a level of 50 mg/kg produced a positive effect on the level of hemoglobin, RBC, WBC, and platelet count. Quercetin also attenuated the hematological parameters that were observed in rats with furan- and cadmium-induced toxicity (Alam et al., 2017; Donmez et al., 2019). This might be due to antioxidant and anti-inflammatory effects of quercetin (Karimi et al., 2021).

Nitrogen balance is investigated in animals or humans to sort out the balance between nitrogen intake and nitrogen excretion from the body. It is an important part of the nutrition research and mechanism to assess an individual’s protein metabolism and overall nutritional status. For this purpose, investigation of nitrogen balance had been performed in this study, and it showed that rats that fed on HOT_1_ diet had shown positive nitrogen balance; however, a non-significant result was observed in rats that fed on HU and HOT_3_ diets. The groups that showed positive nitrogen balance could be due to the increase in their feed intake, CP intake, and digestibility. These parameters were not positive in HOT_2_ resulting in negative nitrogen balance, as shown in Table 14. Essential oils in onion and garlic reduce ruminal NH3-N concentration and increase protein availability in the intestine. Therefore, when protein deamination stopped in the rumen, the ammonia level decreased, allowing ruminants to have higher amino acid availability in the small intestine. Onions have free radical scavenging and anti-inflammatory activity that restricts degenerative changes in the body. Morshedy et al. (2021) provided rocket seed oil (*Eruca sativa* Mill.) and wheat germ oil to rabbits. A mixture of both oils enhanced nitrogen absorption and reduced nitrogen excretion. Quercetin and diallyl disulfide in the onion increase the digestion of protein and promote the growth of friendly microbes in the gut. Kamruzzaman et al. (2011) also reported the positive effect of garlic on nitrogen retention. This effect could be due to the upregulation of the proteolytic enzyme activity in sheep’s rumen and inhibition of ammonia nitrogen deamination. Similarly, according to the work of Nisa et al. (2006), the slower release of N attached to fiber synchronized with fiber fermentation and its utilization by the ruminal microbes, preventing loss. Fiber facilitates the fermentation activities in the gut. It also produces short-chain fatty acids (SCFAs) that provide energy and strength to the intestinal lining for better digestion and absorption. Herbal supplements may retain more nitrogen in the gut and facilitate protein digestion by enhancing the activity of the protease. This could be possible due to the antibacterial activity of the herbal supplement. The higher level of 1.5 kg/ton has shown a positive nitrogen balance compared with other treatments (Saleh et al., 2018). So onion and quercetin regulated the protein metabolism and showed a positive influence on the retention of protein in the body.

## 5 Conclusion

The results showed that HOT_2_, a diet containing onion powder (14.84 g/100 g feed) corresponding to the quercetin content of 0.100 mg/kg, had shown strong anti-hyperuricemia potential which generated a healthy and favorable effect on the serum and hematological traits. Rats that fed on HOT_1_ (11.13 g/100 feed) diet corresponding to the quercetin content of 0.075 mg/kg had shown promising effects on nutrient intake and digestibility, feed intake, body weight, and nitrogen balance in hyperuricemia-induced male Wistar albino rats. Therefore, onion has a protective and therapeutic potential for the cure of various ailments, and it might be used for the occurrence and treatment of hyperuricemia and related disorders.

## Data Availability

The raw data supporting the conclusion of this article will be made available by the authors, without undue reservation.
